# Chemokine CXCL10 and Incident Stroke Risk in the Northern Manhattan Study

**DOI:** 10.1007/s12975-026-01498-7

**Published:** 2026-10-02

**Authors:** Mohammad Nafeli Shahrestani, Christian Agudelo, Hannah Gardener, Emir Veledar, Jose A. Santiago, Marco R. Di Tullio, David Della-Morte, Botagoz Aimagambetova, Jose Gutierrez, Mady Hornig, Mitchell S. V. Elkind, Hyun Song, Clinton B. Wright, Sarah E. Tom, Tatjana Rundek

**Affiliations:** 1 Department of Neurology, Miller School of Medicine, University of Miami, Miami, FL USA; 2 Evelyn F. McKnight Brain Institute, Miller School of Medicine, University of Miami, 1120 NW 14 Street, CRB 1371, Miami, FL 33136 USA; 3 Department of Medicine, Vagelos College of Physicians and Surgeons, Columbia University, New York City, NY USA; 4 Department of Biomedicine and Prevention, University of Rome Tor Vergata, Rome, Italy; 5 Department of Neurology, NYC Health and Hospitals/ Elmhurst, New York, United States of America; 6 Feinstein Institutes for Medical Research, Manhasset, NY USA; 7 Department of Neurology, Vagelos College of Physicians and Surgeons, Columbia University, New York City, NY USA; 8 Department of Epidemiology, Mailman School of Public Health, Columbia University, New York City, NY USA; 9 Disorders and Stroke (NINDS), National Institute of Neurological, National Institutes of Health, Bethesda, MD USA

**Keywords:** CXCL10, IP-10, CXCL9, Chemokines, Inflammation, Stroke, Biomarkers, Northern Manhattan Study

## Abstract

Inflammation contributes to cerebrovascular disease, but prospective studies of broad immune marker panels and incident stroke are limited. We aimed to identify plasma immune markers independently associated with incident stroke. Stroke-free Northern Manhattan Study participants aged ≥ 50 years had 60 plasma immune-related proteins measured by Luminex. Least absolute shrinkage and selection operator (LASSO) Cox regression selected markers associated with incident all-cause stroke. Selected markers were tested in Cox models of stroke risk sequentially adjusted for vascular risk, cardiac comorbidities, interleukin-6, interleukin-1β, tumor necrosis factor-α, and white matter hyperintensity volume (WMHV). Ischemic stroke was a secondary outcome, and bootstrap resampling provided internal validation. Among 1175 participants (mean age 70 ± 9 years; 60% women; 67% Hispanic), 130 strokes, 101 (78%) ischemic, occurred over a median of 14 years. LASSO selected only C-X-C motif chemokine ligands 9 and 10 (CXCL9 and CXCL10). Each 1-standard deviation greater CXCL10 was associated with 20% greater all-cause stroke risk after adjustment for vascular risk, cardiac comorbidities, and inflammatory cytokines (HR 1.20; 95%CI 1.04–1.38). The association persisted after WMHV adjustment (HR 1.18; 95%CI 1.02–1.37), was similar for ischemic stroke (HR 1.19; 95%CI 1.01–1.41), and was stable across bootstrap resamples. CXCL9 was not independently associated with stroke. In a multiethnic urban aging cohort followed prospectively for over a decade, baseline plasma CXCL10, also known as interferon-γ–induced protein 10 (IP-10), was independently associated with incident stroke. CXCL10 may mark interferon-γ–responsive immune activity linked to cerebrovascular vulnerability. Whether CXCL10 signals a modifiable pathway for stroke prevention warrants investigation.

**Supplementary Information:**

The online version contains supplementary material available at https://doi.org/10.1007/s12975-026-01498-7.

## Introduction

Stroke remains a leading cause of mortality and long-term disability worldwide [1]. Stroke risk is commonly estimated with validated prediction tools based on traditional factors, including age, blood pressure, diabetes mellitus, smoking, and atrial fibrillation [2]. Although vascular and lifestyle factors explain much of the population-attributable risk, predictions based on these factors alone remain incomplete, suggesting that additional biological pathways contribute to stroke susceptibility [1]. Inflammation is recognized as a key mechanism in cerebrovascular disease, yet how to capture inflammatory biology in a clinically actionable way remains uncertain [1, 3]. This gap underscores the need for accessible and potentially modifiable immune markers of stroke risk.

Innate and adaptive immune pathways contribute to cerebrovascular disease through endothelial activation, atherosclerotic plaque formation, thrombo-inflammation, and small vessel injury [3]. Cytokine and chemokine signaling modulates leukocyte trafficking, platelet activation, and blood–brain barrier integrity, linking systemic immune tone to neurovascular vulnerability [4]. Chronic low-grade inflammation in older adults may amplify these processes [5]. Because many immune mediators are produced by circulating leukocytes and the vascular endothelium, blood-based markers offer an accessible readout of immune activation relevant to vascular injury. When the blood–brain barrier is disrupted, peripheral signals may also reflect neurovascular inflammation [4, 6].

Prospective studies linking circulating inflammation to stroke have historically focused on a few candidate markers, most commonly high-sensitivity C-reactive protein (hsCRP) and interleukin-6 (IL-6) [7, 8]. Large-scale proteomic platforms have since extended this work beyond individual candidate markers [9]. Aptamer-based and proximity extension assays have profiled hundreds to thousands of plasma proteins in community-based cohorts and identified reproducible associations with incident stroke, led by markers of cardiac wall stress and tissue injury [10–14]. The number of proteins tested in parallel, however, imposes a stringent statistical threshold that immune mediators of modest effect size may not cross. Immune pathways relevant to stroke risk therefore remain incompletely characterized. Focused immune profiling reduces this multiple-testing burden and, paired with principled data reduction, can identify markers that are both biologically interpretable and clinically actionable.

The Northern Manhattan Study (NOMAS) is a well-phenotyped prospective cohort with over two decades of follow-up, originally designed to investigate stroke incidence and risk factors in a multiethnic metropolitan U.S. population [15]. In this cohort, circulating immune markers have been linked to extracranial and intracranial large artery stenosis, carotid atherosclerosis, small vessel disease, and cognitive outcomes [16–19]. Previous proteomic studies of incident stroke were conducted in European cohorts and in U.S. cohorts of White and Black participants, with limited representation of Hispanic adults. Immune-focused profiling in a multiethnic cohort with substantial Hispanic representation is lacking. We hypothesized that a broad panel of circulating immune-related proteins spanning inflammatory, regulatory, and coagulation-linked pathways would identify markers independently associated with incident all-cause stroke.

## Methods

### Study population and design

NOMAS is a community-based cohort that enrolled 3298 individuals aged ≥ 40 years residing in Northern Manhattan, New York City, using random-digit dialing [20]. Between 2003 and 2008, a subset of participants and 199 household members were enrolled in the MRI substudy. The initial sample for this study comprised the 1290 substudy participants who were aged ≥ 50 years, stroke-free at enrollment, and without contraindications to MRI.

**Fig. 1:**
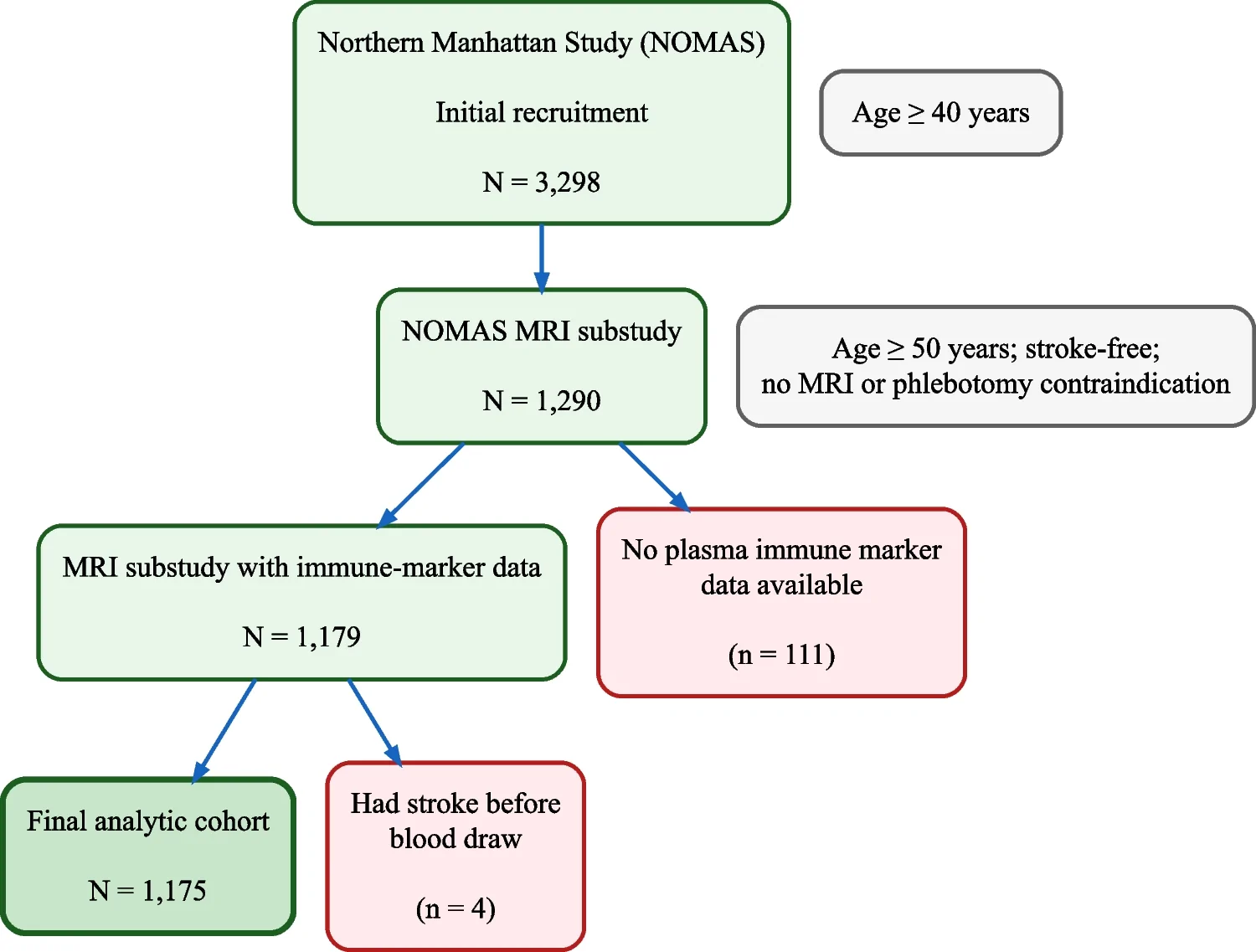
Flow diagram of participant selection

Of these, 1179 had valid immunoassay results, and the 1175 who remained stroke-free until phlebotomy formed the analytic sample (Fig. 1). Reporting followed the Strengthening the Reporting of Observational Studies in Epidemiology (STROBE) guidelines.

### Immune biomarkers

Immune markers were measured at the MRI substudy visit (2003–2008), which served as baseline for this analysis. Fasting plasma was aliquoted and stored at − 80 °C until assay. Sixty immune-related proteins (Supplementary Table 1) were measured in duplicate with a customized magnetic bead–based 60-plex immunoassay (Affymetrix/eBioscience, Thermo Fisher Scientific) on the Luminex 200™ platform (Luminex Corporation, Austin, TX), as previously described [17]. Coded samples were randomized across plates with balanced representation of study variables to minimize batch effects.

The median fluorescence intensity (MFI) of each analyte-specific bead set was acquired with xPONENT software (build 3.1.971.0) with doublet discriminator gating of 5,000–25,000, and duplicate MFIs were averaged. Under a prespecified quality control protocol, plates with a mean intra-assay coefficient of variation (CV) ≥ 15% were rejected, samples with > 20% of analyte CVs exceeding 25% or a mean per-sample CV > 25% were flagged for rerun, and wells with bead counts < 30 were flagged and accepted only if summed counts across both wells exceeded 50. MFIs above the reliable detection range (> 25,000) were excluded. A pooled human plasma control on every plate monitored interassay performance. Analyses used averaged MFI values rather than derived concentrations to avoid bias from extrapolation outside the calibrated standard curve range [21].

### Stroke assessment

Participants were contacted annually by telephone to identify interim neurologic symptoms, new stroke diagnoses, hospitalizations, and deaths, supplemented by ongoing hospital surveillance of admissions and discharges. Suspected strokes triggered medical record review and in-person assessment. Stroke was defined by the World Health Organization criteria as rapidly developing focal or global neurologic deficits lasting > 24 h or leading to death, with no apparent nonvascular cause. Strokes were classified as ischemic infarction, intracerebral hemorrhage, subarachnoid hemorrhage, or unclassified when information was insufficient. Two neurologists classified events independently, and a third neurologist resolved disagreements. The primary outcome was the first incident stroke of any type [20]. Follow-up time extended from blood draw to the first incident stroke, with censoring at death or last follow-up.

### Covariates

At enrollment, trained research assistants collected information on sociodemographic characteristics, vascular risk factors, lifestyle behaviors, and medical history, and participants underwent physical and neurological examinations.

Race and ethnicity (Hispanic, non-Hispanic Black, non-Hispanic White, and Other) were self-reported using questions modeled after the U.S. Census and conforming to Directive 15 definitions [22].

Vascular risk factors and cardiovascular comorbidities, including congestive heart failure (CHF), myocardial infarction (MI), atrial fibrillation (AF), and peripheral vascular disease, were ascertained with standardized questionnaires adapted from the Behavioral Risk Factor Surveillance System [23]. Hypertension was defined as systolic blood pressure ≥ 140 mm Hg, diastolic blood pressure ≥ 90 mm Hg, self-reported hypertension, or antihypertensive medication use. Diabetes mellitus was defined as fasting blood glucose ≥ 126 mg/dL, self-reported diabetes, or glucose-lowering medication use.

Smoking status was classified as current (within the past year), former, or never. Leisure-time physical activity over the preceding two weeks was assessed with National Health Interview Survey questionnaires, and moderate-to-heavy physical activity was defined as at least one vigorous activity. Moderate alcohol consumption was defined as more than one drink per month and no more than two drinks per day.

At the phlebotomy visit, waist and hip circumferences were measured at the umbilicus and the greater trochanters to calculate the waist-to-hip ratio [24]. Blood pressure was measured twice with an automated sphygmomanometer after a 10-min rest and averaged [25]. Fasting blood samples were assayed for glucose, total cholesterol, and high-density and low-density lipoprotein cholesterol.

Vascular risk burden was summarized with the global vascular risk score (GVRS), which combines demographic, behavioral, anthropometric, and clinical risk factors [26]. The GVRS was derived and validated in NOMAS to estimate the long-term risk of major vascular events, including ischemic stroke, and outperforms the Framingham risk score in this population [27].

White matter hyperintensity volume was quantified on T2-weighted fluid-attenuated inversion recovery images acquired on a 1.5 T scanner (Philips Intera) with a semiautomated segmentation method and normalized to total cranial volume (WMHV), as previously described [16]. Blood was drawn on the day of MRI in 1139 (92%) participants, and the mean interval between phlebotomy and MRI was 0.7 (standard deviation [SD] 13) days.

### Statistical analysis

Baseline characteristics were summarized with descriptive statistics (Table 1). Cox proportional hazards regression with a least absolute shrinkage and selection operator (LASSO) penalty was applied to the 60 z-standardized immune markers to select markers associated with time to all-cause stroke. The penalty was chosen by fivefold cross-validation of the partial likelihood deviance, and markers with nonzero coefficients at the penalty minimizing cross-validated deviance (λ_min_) were retained. This penalized selection served as the data-reduction step. The two retained markers, C-X-C motif chemokine ligand 9 (CXCL9) and C-X-C motif chemokine ligand 10 (CXCL10), were then tested in the Cox models described below. Because selection and testing used the same data, p values from these models do not account for the selection step and are reported as nominal.

**Table 1:** Baseline cohort characteristics by subsequent stroke subtype

Variable	Entire cohort (*N* = 1175)	Stroke-free (*N* = 1045)	All Stroke (*N* = 130)	Ischemic Stroke (*N* = 101)	Nonischemic Stroke* (*N* = 29)
CXCL9, MFI (median [IQR])	329 [248–493]	324 [244–477]	385 [270–584]	377 [278–561]	464 [259–637]
CXCL10, MFI (median [IQR])	1029 [730–1441]	1016 [727–1434]	1101 [827–1504]	1112 [867–1476]	1110 [722–1753]
Demographics					
Age at blood draw, years, mean ± SD	70.3 ± 8.9	70 ± 8.9	73.7 ± 8.5	74.1 ± 8.9	72.7 ± 7.5
Sex, male, n (%)	468 (40%)	422 (40%)	46 (35%)	37 (37%)	9 (31%)
Race and ethnicity, n (%)					
Hispanic	784 (67%)	696 (67%)	88 (68%)	70 (69%)	18 (62%)
Non-Hispanic White	171 (15%)	151 (14%)	20 (15%)	15 (15%)	5 (17%)
Non-Hispanic Black	196 (17%)	174 (17%)	22 (17%)	16 (16%)	6 (21%)
Other	24 (2%)	24 (2%)	0 (0%)	0 (0%)	0 (0%)
Global vascular risk score, mean ± SD	8.6 ± 0.9	8.6 ± 0.9	9.0 ± 0.8	9.0 ± 0.8	9.0 ± 0.7
Atrial fibrillation, n (%)	28 (2%)	20 (2%)	8 (6%)	6 (6%)	2 (7%)
Congestive heart failure, n (%)	34 (3%)	24 (2%)	10 (8%)	6 (6%)	4 (14%)
Myocardial infarction, n (%)	35 (3%)	32 (3%)	3 (2%)	3 (3%)	0 (0%)
WMHV, % TCV, median [IQR]	0.37 [0.21–0.76]	0.33 [0.20–0.68]	0.60 [0.25–0.89]	0.61 [0.30–0.81]	0.77 [0.25–1.25]

*CXCL9* C-X-C motif chemokine ligand 9, *CXCL10* C-X-C motif chemokine ligand 10, *MFI* median fluorescence intensity, *IQR* interquartile range, *SD* standard deviation, *WMHV* white matter hyperintensity volume, *TCV* total cranial volume

^*^This group included hemorrhagic stroke events (16, intracerebral and subarachnoid) and unclassified stroke events (13)

Because chemokine distributions were right-skewed, bivariate associations with demographic, clinical, inflammatory, and imaging covariates were assessed with Spearman rank correlations, Mann–Whitney U tests, and Kruskal–Wallis tests with Dunn post hoc comparisons (Supplementary Table 2 and Supplementary Figure 1). The contribution of measured risk factors to variance in circulating CXCL10 was quantified with a multivariable linear model and relative importance analysis (Supplementary Methods). Stroke-free survival by median and quartile categories of each chemokine was estimated with the Kaplan–Meier method and compared with log-rank tests.

Each chemokine was entered separately into Cox proportional hazards models as a z-standardized, untransformed MFI value, with sequential adjustment. Model 1 included age, sex, and race and ethnicity. Model 2 included the GVRS. Model 3 added history of AF, MI, and CHF. Model 4 added IL-6, interleukin-1β (IL-1β), and tumor necrosis factor-α (TNF-α) to test independence from established inflammatory pathways [3, 7, 8, 28]. Model 5 added WMHV. Both chemokines were also entered together in Models 4 and 5. Collinearity was assessed with variance inflation factors and the condition number of the scaled model matrix [29]. Departures from linearity were assessed as described in the Supplementary Methods.

Effect modification by age, sex, and interferon-γ (IFN-γ) was tested with chemokine × modifier interaction terms in GVRS-adjusted models of all-cause stroke. The CXCL9 × CXCL10 interaction was tested in Model 2 containing both chemokines.

Sensitivity analyses restricted the outcome to ischemic stroke, additionally adjusted Model 5 for estimated glomerular filtration rate (eGFR) and for hsCRP in the subset with available data, and excluded strokes within the first 1 and 2 years of follow-up.

The stability of fully adjusted (Model 5) all-cause hazard ratios for CXCL9 and CXCL10 was evaluated with nonparametric bootstrap resampling (2000 iterations). To account for data-driven selection, the entire LASSO procedure was repeated in each of 2000 bootstrap resamples. The inclusion frequency of each marker was the proportion of resamples in which it was selected. Each of the 60 markers was also tested in a separate Cox model adjusted for age, sex, and race and ethnicity, with Benjamini–Hochberg false discovery rate (FDR) correction across all 60 tests (Supplementary Methods).

To test whether CXCL10 improves stroke prediction beyond established clinical risk factors, Model 4 was compared with and without CXCL10 using the likelihood ratio test, Harrell’s C-index, time-dependent areas under the receiver operating characteristic curve (AUCs), and reclassification metrics. Model 4 rather than Model 5 was used because WMHV requires volumetric segmentation of brain MRI and is not available in routine clinical practice.

Analyses were conducted in R version 4.5.0 (RStudio 2025.05.0 + 496) with the tidyverse, glmnet, survival, boot, parallel, and relaimpo packages.

## Results

The baseline cohort characteristics are presented in Table 1. Among 1175 stroke-free participants (mean age 70 ± 9 years; 60% women; 67% Hispanic), 130 incident strokes occurred over a median follow-up of 14 (IQR 10–16) years. Of these, 101 (78%) were ischemic, 11 (8%) were intracerebral hemorrhages, 5 (4%) were subarachnoid hemorrhages, and 13 (10%) were unclassified.

Hypertension was present in 897 (76%) participants, diabetes mellitus in 240 (20%), and peripheral vascular disease in 141 (12%). Nearly half (48%) had never smoked, 96 (8%) engaged in moderate-to-heavy physical activity, and 367 (31%) reported moderate alcohol consumption.

**Fig. 2:**
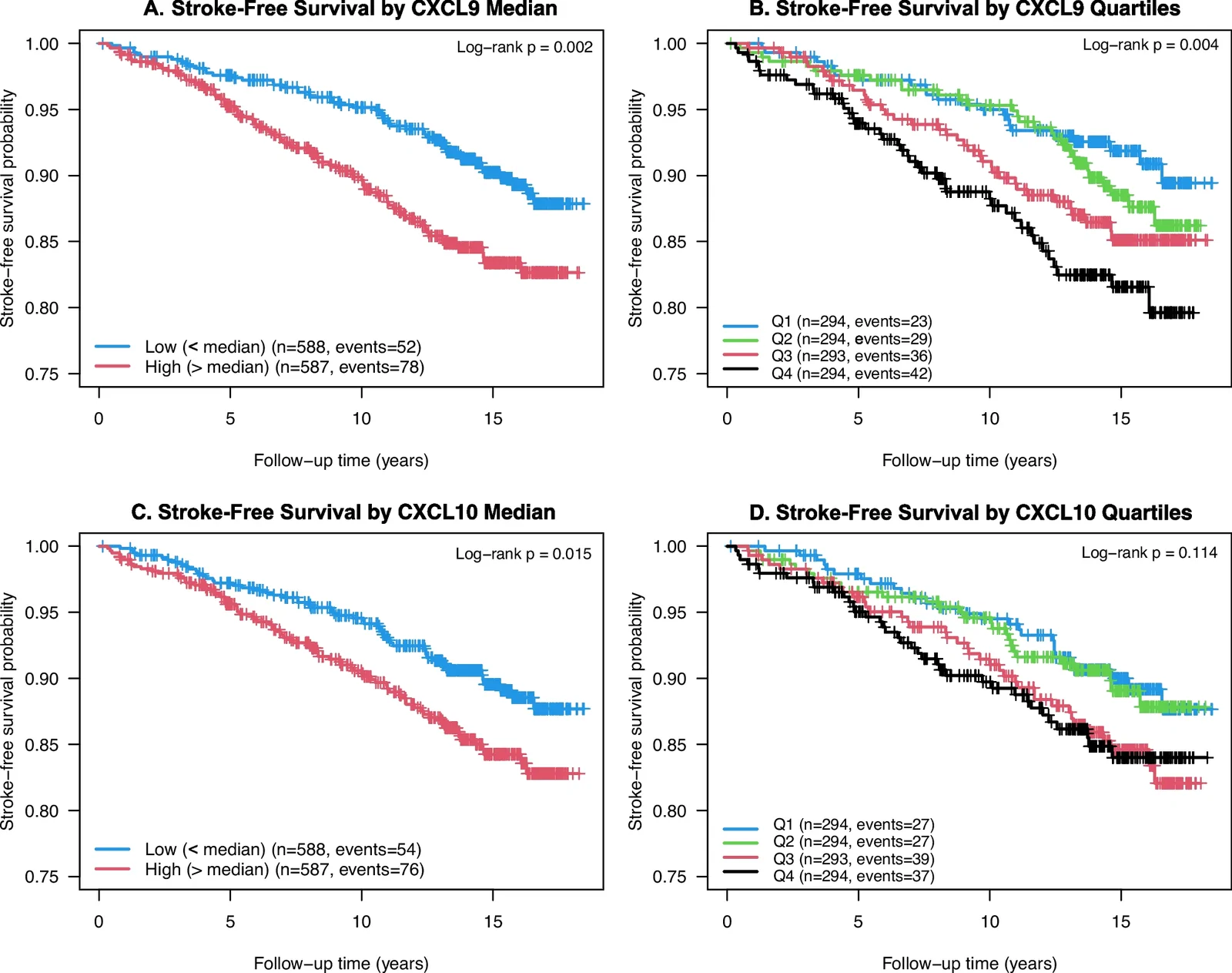
Kaplan–Meier curves for all-cause incident stroke according to baseline plasma CXCL9 and CXCL10 levels (*N* = 1175). Panels A and B show stroke-free survival by median and quartile categories of CXCL9, respectively. Panels C and D show the same for CXCL10. Chemokine levels are expressed as median fluorescence intensity (MFI). Tick marks indicate censoring, and p values are from log-rank tests. CXCL9 = C-X-C motif chemokine ligand 9; CXCL10 = C-X-C motif chemokine ligand 10; Q = quartile

Among the 60 immune markers screened, LASSO selected only CXCL9 and CXCL10 and shrank all other coefficients to zero (Supplementary Table 1). In Kaplan–Meier analyses (Fig. 2), participants with above-median CXCL9 or CXCL10 had greater cumulative stroke incidence (log-rank *p* = 0.002 and *p* = 0.015, respectively). Stroke incidence differed across CXCL9 quartiles (log-rank *p* = 0.004), with progressively shorter stroke-free survival in upper quartiles, but not across CXCL10 quartiles (log-rank *p* = 0.11).

CXCL9 was more strongly correlated than CXCL10 with age (*ρ* = 0.32 versus 0.14) and with GVRS (*ρ* = 0.25 versus 0.09; Supplementary Table 2). CXCL9 and CXCL10 were moderately correlated with each other (Spearman *ρ* = 0.63; 95% CI 0.59–0.66), whereas correlations of both chemokines with IL-6, IL-1β, TNF-α, hsCRP, and WMHV were weak (all |ρ| ≤ 0.22; Supplementary Figure 1). Demographic, clinical, renal, and imaging risk factors together explained 5.3% of the variance in circulating CXCL10, and no single factor explained more than 1.1% (Supplementary Table 6).

In multivariable Cox models, each 1-SD greater CXCL10 was associated with 21% greater all-cause stroke risk after adjustment for GVRS and cardiac comorbidities (Model 3; HR 1.21; 95% CI 1.06–1.39). The association persisted after further adjustment for IL-6, IL-1β, and TNF-α (Model 4; HR 1.20; 95% CI 1.04–1.38) and for WMHV (Model 5; HR 1.18; 95% CI 1.02–1.37; Table 2). In Model 1, the CXCL10 hazard ratio (1.19) was comparable to that of approximately 2.6 additional years of age (HR 1.07 per year; 95% CI 1.05–1.10). CXCL9 was not associated with all-cause or ischemic stroke in any model (all *p* > 0.05). When both chemokines were entered together, CXCL10 remained associated with stroke (Model 4; HR 1.20; 95% CI 1.03–1.40), whereas CXCL9 was not (HR 0.98; 95% CI 0.78–1.24). In Model 5 containing both chemokines, all variance inflation factors were below the conventional threshold of 5 (maximum 2.6; CXCL9, 1.25; CXCL10, 1.27), and the condition number was 4.0 against a threshold of 30, indicating no collinearity [29].

**Table 2:** Associations of CXCL9 and CXCL10 with all-cause and ischemic stroke risk (*N* = 1175)

			All-cause (*N* = 130)			Ischemic (*N* = 101; 78%)		
	Adjustment	Variable*	HR^†^	95% CI	P value	HR^†^	95% CI	P value
Model 1	Demographics^‡^	CXCL9	1.13	0.98–1.31	0.095	1.14	0.98–1.34	0.089
		CXCL10	1.19	1.03–1.36	0.016	1.19	1.01–1.39	0.034
Model 2	GVRS	CXCL9	1.11	0.96–1.27	0.15	1.13	0.97–1.31	0.12
		CXCL10	1.20	1.05–1.37	0.007	1.21	1.04–1.40	0.016
Model 3	GVRS + AF + CHF + MI	CXCL9	1.11	0.96–1.27	0.13	1.12	0.96–1.31	0.16
		CXCL10	1.21	1.06–1.39	0.005	1.21	1.03–1.41	0.017
Model 4	GVRS + AF + CHF + MI + CIM^§^	CXCL9	1.09	0.90–1.33	0.36	1.11	0.89–1.37	0.38
		CXCL10	1.20	1.04–1.38	0.010	1.20	1.02–1.41	0.025
Model 5	GVRS + AF + CHF + MI + CIM^§^ + WMHV	CXCL9	1.07	0.87–1.32	0.51	1.09	0.88–1.37	0.41
		CXCL10	1.18	1.02–1.37	0.022	1.19	1.01–1.41	0.030

*CXCL9* C-X-C motif chemokine ligand 9, *CXCL10* C-X-C motif chemokine ligand 10, *HR* hazard ratio, *CI* confidence interval, *GVRS* global vascular risk score, *AF* atrial fibrillation, *CHF* congestive heart failure, *MI* myocardial infarction, *CIM* circulating immune marker, *WMHV* white matter hyperintensity volume, *MFI* median fluorescence intensity

^*^Each chemokine was tested in a separate model

^†^Hazard ratio per 1-standard deviation (SD) greater chemokine level. One SD corresponded to an MFI of 480 for CXCL9 and 776 for CXCL10

^‡^Model 1 was adjusted for age at blood draw, sex, and race and ethnicity (Hispanic, non-Hispanic Black, non-Hispanic White, or Other [*n* = 24, 2%])

^§^The circulating immune markers included in the model were interleukin-6 (IL-6), interleukin-1β (IL-1β), and tumor necrosis factor-α (TNF-α)

Restricted cubic spline analyses supported approximately linear associations for both chemokines (Supplementary Figures 2 and 3; Supplementary Tables 3 and 4; Supplementary Results). There was no evidence of effect modification by age, sex, or IFN-γ, or of interaction between CXCL9 and CXCL10 (all p for interaction > 0.10).

In sensitivity analyses, the CXCL10 association was consistent for ischemic stroke (Model 5; HR 1.19; 95% CI 1.01–1.41) and persisted after additional adjustment for eGFR (HR 1.21; 95% CI 1.04–1.40; *p* = 0.012) and for hsCRP among the 718 participants with available data (HR 1.19; 95% CI 1.07–1.33). The fully adjusted association persisted after excluding strokes within the first year (121 events; HR 1.17; 95% CI 1.001–1.36) but was no longer significant after a 2-year exclusion (113 events; HR 1.12; 95% CI 0.94–1.33).

In bootstrap internal validation (2000 resamples), the CXCL10 association remained stable (median all-cause HR 1.18; 95% percentile interval 1.001–1.35), whereas CXCL9 showed no consistent association (median HR 1.07; 95% percentile interval 0.84–1.20).

When the entire LASSO procedure was repeated across 2000 bootstrap resamples, CXCL10 was the most frequently selected of the 60 markers (76% of resamples), whereas CXCL9 was selected in 55% (Supplementary Table 7). In separate demographics-adjusted models of each of the 60 markers, CXCL10 had the greatest hazard ratio and was the only marker with nominal *p* < 0.05 (HR 1.19; *p* = 0.017). This association did not remain significant after FDR correction across 60 tests (*q* = 0.90).

Adding CXCL10 to the clinical-inflammatory base model (Model 4) improved model fit (likelihood ratio χ^2^ = 5.39, df = 1, *p* = 0.020). Harrell’s C-index increased from 0.687 to 0.696, and time-dependent AUCs increased from 0.675 to 0.688 at 5 years, from 0.738 to 0.751 at 10 years, and from 0.741 to 0.748 at 14 years. At 10 years, the continuous net reclassification improvement was 0.115 (95% CI − 0.020 to 0.239; *p* = 0.090), and the integrated discrimination improvement was 0.0048 (95% CI − 0.0017 to 0.0187; *p* = 0.178; Supplementary Table 5).

## Discussion

In this prospective multiethnic urban cohort of middle-aged to older adults followed for a median of 14 years, an agnostic screen of 60 circulating immune markers prioritized two closely related IFN-γ–inducible chemokines, CXCL9 and CXCL10. Greater baseline CXCL10 was consistently associated with incident all-cause and ischemic stroke, independent of vascular risk factors, cardiovascular comorbidities, and established inflammatory markers.

Over the 14-year follow-up, a 1-SD greater CXCL10 corresponded to approximately 2 additional strokes per 100 participants. Stroke arises from multifactorial, cumulative vascular injury, and no single circulating marker is expected to account for individual susceptibility in isolation [1]. Adding CXCL10 to the clinical-inflammatory model improved model fit, but the gain in discrimination was modest, and reclassification did not improve significantly. These data do not establish clinical utility for CXCL10 as a standalone predictor, a pattern well recognized when a single biomarker is added to an established prediction model [28]. The prognostic value of chemokines may lie in their incorporation into multiparametric approaches that integrate clinical, imaging, and laboratory explorations [30]. CXCL10 may also help identify heightened immunovascular vulnerability in the presence of vascular risk factors and provide insight into stroke pathogenesis.

Genetic evidence supports a potential causal role of CXCL10 in cerebrovascular disease. A proteogenomic analysis of 3281 plasma proteins, using data from over 500,000 individuals, identified CXCL10 as the only protein affected by IL-6 signaling whose genetically proxied levels were associated with atherosclerotic disease [31]. A Mendelian randomization study of 41 inflammatory cytokines linked genetically predicted CXCL10 to both ischemic stroke and intracerebral hemorrhage [32]. In the Prospective Epidemiological Study of Myocardial Infarction (PRIME), which followed 9771 initially healthy middle-aged European men for 10 years, greater baseline CXCL10 was independently associated with ischemic stroke [33]. Greater circulating CXCL10 has also been reported in carotid atherosclerosis, at stroke admission, and in relation to poorer functional outcomes [33–37]. The present study extends this evidence to men and women from multiple racial and ethnic groups in a cohort with a longer observation period (median 14 years vs. 10 years in PRIME). In bootstrap resampling, CXCL10 was the marker most frequently selected by LASSO, indicating that its selection was robust to sampling variability. Its fully adjusted hazard ratio also remained stable across resamples, providing internal validation of the adjusted analysis.

Recent large-scale proteomic studies of incident stroke identified markers of cardiac wall stress and tissue injury rather than chemokines [10–14], including studies in which CXCL10 was included in the assay but was not reported among the proteins associated with incident stroke [10, 13, 14]. Correction for thousands of simultaneous comparisons in these platform-wide screens may obscure a modest chemokine signal. Our screen was restricted to immune markers, and our cohort has substantial Hispanic representation, which is unique among these prior studies. These differences may explain why CXCL10 did not emerge in these analyses.

The association was consistent for ischemic stroke, in line with the PRIME and proteogenomic findings [31, 33]. A recent multi-omics study also identified an inflammatory axis involving CXCL10 as a master regulator of microglial programmed cell death in ischemic stroke [38]. The association persisted after adjustment for WMHV, an established marker of cerebral small vessel disease [39], and after exclusion of events in the first year of follow-up. These findings, together with the genetic evidence [31, 32], argue against CXCL10 being merely a byproduct of preexisting subclinical cerebrovascular disease. The association was lost, however, after exclusion of the first 2 years, likely reflecting reduced power from the substantial loss of events.

Our findings also underscore the biological heterogeneity of inflammation in stroke risk. Acute-phase reactants and pleiotropic cytokines primarily reflect systemic inflammation, whereas chemokines propagate neuroinflammation via recruitment of activated immune cells to sites of inflammation [7, 8]. Consistent with this distinction, the CXCL10 association changed little after adjustment for IL-6, IL-1β, TNF-α, and hsCRP. One possibility is that CXCL10 elevation is a byproduct of an established stroke risk factor. The present data argue against this. The CXCL10–stroke association persisted after adjustment for demographics and established clinical and imaging risk factors, including vascular burden, cardiac comorbidities, and renal function. Together, these risk factors explained only 5% of the variance in circulating CXCL10 (Supplementary Table 6). CXCL10 therefore appears to capture immune activity relevant to cerebrovascular vulnerability rather than a downstream readout of established vascular risk.

A plausible mechanism is signaling through C-X-C motif chemokine receptor 3 (CXCR3). CXCL9 and CXCL10 are canonical IFN-γ–inducible ligands of CXCR3, a receptor expressed on immune, vascular, and central nervous system cells [40]. CXCR3 signaling directs T-cell migration, drives chronic inflammation, amplifies endothelial activation, and sustains thrombo-inflammatory interactions [41, 42]. It has also been implicated in atherosclerosis and plaque vulnerability [43], and IFN-γ signaling contributes to endothelial dysfunction, oxidative stress, and vascular remodeling [44, 45]. Although CXCL9 and CXCL10 share a receptor and are often co-expressed, they are not functionally redundant and differ in tissue expression, receptor binding, and downstream effects [46]. These differences may explain why CXCL10 retained an independent association with stroke after accounting for CXCL9, despite the correlation between the two markers (Supplementary Figure 1). CXCL9 was more strongly correlated with age and GVRS and was not associated with stroke after adjustment for demographics. Prior NOMAS findings linked CXCL9, but not CXCL10, to white matter disease burden [16]. CXCL9 may therefore reflect chronic small vessel injury related to aging rather than processes that precipitate overt stroke.

The CXCL10–stroke association was approximately linear with no evidence of a threshold effect, indicating that risk varies continuously across the full range of CXCL10 levels. Because lower levels correspond to lower risk, these findings raise the possibility that reducing CXCL10 could be protective.

Anti-inflammatory therapies such as canakinumab and colchicine have shown that inflammatory pathways are modifiable contributors to cardiovascular risk [47, 48]. CXCL10 itself may be pharmacologically modifiable. Peroxisome proliferator-activated receptor-γ agonists suppress its expression in human endothelial cells, and atorvastatin reduces circulating CXCL10 [49–51]. Monoclonal antibodies against CXCL10, including eldelumab and NI-0801, have been tested in immune-mediated conditions, although their relevance to stroke prevention is unknown [52–54].

Strengths of this study include a multiethnic community-based cohort with substantial vascular burden, extended follow-up, rigorous stroke adjudication, and internal validation. Several limitations should be considered. A single biomarker measurement cannot distinguish stable from transient immune activation, and within-person variability of these chemokines over time is unknown. The small numbers of hemorrhagic and ischemic subtype events precluded subtype-specific analyses, and null interaction findings should be interpreted cautiously given limited power. Immune markers were measured only in participants who remained stroke-free from enrollment through the MRI substudy, and selection toward a stroke-resilient subgroup cannot be excluded. Cardiac comorbidities were ascertained at cohort enrollment rather than at immune profiling. Marker selection and hypothesis testing were performed in the same dataset, and the reported p values do not account for the uncertainty of the selection step. In the screen of all 60 markers, the CXCL10 association did not withstand correction for multiple testing. CXCL10 was nonetheless the marker most frequently selected across bootstrap resamples, and its association with stroke is concordant with prospective and genetic evidence from independent populations [31–33]. External replication remains necessary to confirm this finding. Although adjustment was extensive, CXCL10 might yet be explained by a risk factor this study did not measure, and residual confounding cannot be excluded. Finally, the observational design precludes causal inference, and elevated CXCL10 may reflect upstream immune activation rather than a direct role in cerebrovascular injury.

## Conclusion

In a multiethnic urban cohort followed for over a decade, greater circulating CXCL10 was associated with incident stroke. This association was independent of established vascular risk factors, cardiac comorbidities, commonly measured inflammatory cytokines, kidney function, and WMHV. CXCL10 was also the most frequently selected of 60 immune markers across bootstrap resamples, and its association with stroke is concordant with prospective and genetic evidence from independent populations. This IFN-γ–inducible chemokine may therefore capture an immunovascular dimension of cerebrovascular vulnerability not explained by vascular burden. External replication and integration of CXCL10 with vascular and imaging phenotypes are the next steps. Because CXCL10 is measurable in plasma and potentially modifiable, targeting IFN-γ–responsive chemokine signaling may offer a new avenue to reduce stroke risk and preserve vascular brain health.

## Supplementary Information

Below is the link to the electronic supplementary material.

Supplementary Tables (DOCX 33 KB)

Supplementary Figures (DOCX 731 KB)

Supplementary Methods and Results (DOCX 16 KB)

## Data Availability

The dataset and R scripts used in this study will be made available upon request.

## Declarations

### Data access statement

MNS had full access to all the data in the study and takes responsibility for the integrity of the data and the accuracy of the data analysis.
