# Supplementary material for: Chemokine CXCL10 and Incident Stroke Risk in the Northern Manhattan Study
Source: Transl Stroke Res. 2026 Oct 2;17(5):107. doi: 10.1007/s12975-026-01498-7 (PMC13633176; doi:10.1007/s12975-026-01498-7)
Supplement: Supplementary file 1 [file 12975_2026_1498_MOESM1_ESM.docx]

**Supplementary Table 1.** Plasma Immune Markers Assayed in the 60-plex Luminex Immunoassay and LASSO-Selected Predictors of Incident Stroke

**Panel A. LASSO-Selected Markers**

| **Selected Marker** | **LASSO Coefficient (β)** |
| --- | --- |
| CXCL9 (MIG) | 0.236 |
| CXCL10 (IP-10) | 0.120 |

**Panel B. All Immune Molecules in the 60-plex Immunoassay**

| **Type of Immune Molecule** | **Immune Molecule** |
| --- | --- |
| IL-1 superfamily | IL-1α, IL-1β, IL-1RA, IL-18 |
| IL-12 family | IL-12p40, IL-12p70, IL-23, IL-27 |
| IL-17 family | IL-17A, IL-17F, IL-22 |
| IL-2 family | IL-2, IL-4, IL-7, IL-9, IL-15, IL-21 |
| Th2 type | IL-5, IL-10, IL-13 |
| IL-6 (gp130) cytokine family | IL-6, IL-31, LIF |
| Type I IFN | IFN-α2, IFN-β |
| Type II IFN | IFN-γ |
| TNF superfamily | TNF-α (TNFSF2), TNF-β (TNFSF1), sFasL (TNFSF6), TRAIL (TNFSF10) |
| CC chemokines | CCL2 (MCP-1), CCL3 (MIP-1α), CCL4 (MIP-1β), CCL5 (RANTES), CCL7 (MCP-3), CCL11 (eotaxin) |
| CXC chemokines | CXCL1 (GRO-α), CXCL8 (IL-8), CXCL9 (MIG)†, CXCL10 (IP-10)†, CXCL12α (SDF-1α) |
| PDGF family/VEGF subfamily | PDGF-BB, VEGF-A, VEGF-D |
| Other growth factors | TGF-α, TGF-β, EGF, FGF-basic, HGF, β-NGF |
| Neurotrophic/stimulating factors | BDNF, CSF1 (M-CSF), CSF2 (GM-CSF), CSF3 (G-CSF), SCF |
| Cell adhesion molecules | sICAM-1 (CD54), VCAM-1 (CD106) |
| Serine protease inhibitors | PAI-1 (Serpin E1) |
| Adipose-derived hormones | Leptin, resistin |

†LASSO-selected markers (see Panel A). All other marker coefficients were shrunk to zero at the penalty minimizing cross-validated partial likelihood deviance (λ_min_).

LASSO = least absolute shrinkage and selection operator. LASSO-penalized Cox proportional hazards regression with fivefold cross-validation was applied to z-standardized median fluorescence intensity (MFI) values of all 60 markers. Only CXCL9 and CXCL10 retained nonzero coefficients.

BDNF = brain-derived neurotrophic factor; CCL = C-C motif chemokine ligand; CSF = colony-stimulating factor; CXCL = C-X-C motif chemokine ligand; EGF = epidermal growth factor; FGF-basic = basic fibroblast growth factor; G-CSF = granulocyte colony-stimulating factor; GM-CSF = granulocyte-macrophage colony-stimulating factor; gp130 = glycoprotein 130; GRO-α = growth-regulated oncogene-α; HGF = hepatocyte growth factor; IFN = interferon; IL = interleukin; IL-1RA = interleukin-1 receptor antagonist; IP-10 = interferon-γ–induced protein 10; LIF = leukemia inhibitory factor; MCP = monocyte chemoattractant protein; M-CSF = macrophage colony-stimulating factor; MIG = monokine induced by interferon-γ; MIP = macrophage inflammatory protein; NGF = nerve growth factor; PAI-1 = plasminogen activator inhibitor-1; PDGF-BB = platelet-derived growth factor, BB isoform; RANTES = regulated on activation, normal T cell expressed and secreted; SCF = stem cell factor; SDF-1α = stromal cell-derived factor-1α; Serpin E1 = serpin family E member 1; sFasL = soluble Fas ligand; sICAM-1 = soluble intercellular adhesion molecule-1; TGF = transforming growth factor; Th2 = T helper 2; TNF = tumor necrosis factor; TNFSF = TNF superfamily; TRAIL = TNF-related apoptosis-inducing ligand; VCAM-1 = vascular cell adhesion molecule-1; VEGF = vascular endothelial growth factor.

**Supplementary Table 2.** Associations of CXCL9 and CXCL10 with Demographic and Clinical Covariates

| **Covariate** | **CXCL9** | **CXCL10** | **Statistical test** |
| --- | --- | --- | --- |
| Age | ρ = 0.32 (0.26–0.37), p < 0.001 | ρ = 0.14 (0.08–0.19), p < 0.001 | Spearman correlation |
| Sex (female vs. male) | p = 0.076 | Median [IQR] 1076 [760–1479] vs. 964 [698–1392], p = 0.003 | Mann–Whitney U |
| GVRS | ρ = 0.25, p < 0.001 | ρ = 0.09, p = 0.004 | Spearman correlation |
| Race and ethnicity | χ²(3) = 16.88, p < 0.001† | χ²(3) = 2.88, p = 0.41 | Kruskal–Wallis |
| Myocardial infarction | Median 406 vs. 326, p = 0.008 | p > 0.05 | Mann–Whitney U |
| Atrial fibrillation | p > 0.05 | p > 0.05 | Mann–Whitney U |
| Congestive heart failure | p > 0.05 | p > 0.05 | Mann–Whitney U |

CXCL9 = C-X-C motif chemokine ligand 9; CXCL10 = C-X-C motif chemokine ligand 10; IQR = interquartile range; GVRS = global vascular risk score; ρ = Spearman rank correlation coefficient (95% confidence interval).

†In Dunn post hoc comparisons, CXCL9 levels were greater in Hispanic than in non-Hispanic White participants (p = 0.010). CXCL9 levels were also greater in Hispanic (p < 0.001), non-Hispanic Black (p = 0.001), and non-Hispanic White participants (p < 0.001) than in the Other group.

**Supplementary Table 3.** Associations of Above-Median Versus Below-Median CXCL9 and CXCL10 with Stroke Risk (N = 1175)*

| **Variable** | **Model 1 (adjusted for demographics**)** | | | **Model 2 (adjusted for GVRS)** | | |
| --- | --- | --- | --- | --- | --- | --- |
|  | **HR** | **95% CI** | **P value** | **HR** | **95% CI** | **P value** |
| CXCL9  (above vs. below median) | 1.31 | 0.91–1.88 | 0.14 | 1.31 | 0.92–1.88 | 0.13 |
| CXCL10  (above vs. below median) | 1.33 | 0.94–1.90 | 0.10 | 1.39 | 0.97–1.97 | 0.068 |

HR = hazard ratio; CXCL9 = C-X-C motif chemokine ligand 9; CXCL10 = C-X-C motif chemokine ligand 10; GVRS = global vascular risk score; CI = confidence interval.

*Each chemokine was tested in a separate model.

**Demographics included age at blood draw, sex, and race and ethnicity (Hispanic, non-Hispanic Black, non-Hispanic White, and Other).

**Supplementary Table 4.** Associations of CXCL9 and CXCL10 Quartiles with Stroke Risk (N = 1175)*

| **Variable** | **Model 1 (adjusted for demographics**)** | | | **Model 2 (adjusted for GVRS)** | | |
| --- | --- | --- | --- | --- | --- | --- |
|  | **HR** | **95% CI** | **P value** | **HR** | **95% CI** | **P value** |
| CXCL9 | | | | | | |
| Q2 | 1.14 | 0.66–1.97 | 0.64 | 1.15 | 0.67–2.00 | 0.60 |
| Q3 | 1.26 | 0.74–2.14 | 0.39 | 1.26 | 0.74–2.15 | 0.38 |
| Q4 | 1.58 | 0.93–2.68 | 0.090 | 1.61 | 0.96–2.72 | 0.072 |
| Ordinal (per 1-quartile greater) | 1.16 | 0.98–1.37 | 0.075 | 1.17 | 0.99–1.38 | 0.062 |
| CXCL10 | | | | | | |
| Q2 | 1.10 | 0.64–1.88 | 0.72 | 1.15 | 0.67–1.97 | 0.62 |
| Q3 | 1.46 | 0.89–2.39 | 0.13 | 1.52 | 0.92–2.50 | 0.10 |
| Q4 | 1.34 | 0.81–2.21 | 0.25 | 1.45 | 0.87–2.40 | 0.15 |
| Ordinal (per 1-quartile greater) | 1.12 | 0.95–1.31 | 0.15 | 1.15 | 0.98–1.34 | 0.087 |

HR = hazard ratio; CXCL9 = C-X-C motif chemokine ligand 9; CXCL10 = C-X-C motif chemokine ligand 10; GVRS = global vascular risk score; CI = confidence interval; Q = quartile. The lowest quartile (Q1) is the reference category.

*Each chemokine was tested in a separate model.

**Demographics included age at blood draw, sex, and race and ethnicity (Hispanic, non-Hispanic Black, non-Hispanic White, and Other).

**Supplementary Table 5.** Incremental Discrimination and Reclassification After Adding CXCL10 to the Clinical-Inflammatory Base Model (Model 4)

| **Metric** | **Base Model** | **CXCL10 Model** | **Improvement (95% CI)** | **P value** |
| --- | --- | --- | --- | --- |
| CXCL10 HR per 1-SD | — | 1.20 (1.04–1.38) | — | 0.010 |
| Model fit (LRT) | — | — | χ² = 5.39, df = 1 | 0.020 |
| C-index | 0.687 | 0.696 | +0.009 | — |
| 5-year AUC | 0.675 | 0.688 | +0.014 | — |
| 10-year AUC | 0.738 | 0.751 | +0.013 | — |
| 14-year AUC | 0.741 | 0.748 | +0.007 | — |
| IDI (10-year) | — | — | 0.0048 (−0.0017 to 0.0187) | 0.178 |
| Continuous NRI (10-year) | — | — | 0.115 (−0.020 to 0.239) | 0.090 |

The base model included the global vascular risk score (GVRS), atrial fibrillation, myocardial infarction, congestive heart failure, and circulating inflammatory markers (interleukin-6, interleukin-1β, and tumor necrosis factor-α). CXCL10 was entered as a z-standardized continuous variable (1 SD = 776 MFI). The C-index was estimated using Harrell's concordance statistic. Time-dependent AUCs were estimated using inverse probability of censoring weighting. Model comparison was performed using the likelihood ratio test. Reclassification metrics were computed at the 10-year horizon. AUC = area under the receiver operating characteristic curve; CI = confidence interval; HR = hazard ratio; IDI = integrated discrimination improvement; LRT = likelihood ratio test; MFI = median fluorescence intensity; NRI = net reclassification improvement (continuous/category-free); SD = standard deviation.

**Supplementary Table 6.** Determinants of Variance in Circulating Plasma CXCL10 Levels (N = 1175)

| **Variable** | **Relative importance, %** | **Absolute variance explained, %** |
| --- | --- | --- |
| Sex | 20.4 | 1.09 |
| Age | 19.8 | 1.06 |
| LDL cholesterol | 15.2 | 0.81 |
| Estimated glomerular filtration rate | 14.4 | 0.77 |
| White matter hyperintensity volume | 8.5 | 0.45 |
| HDL cholesterol | 7.9 | 0.42 |
| Race and ethnicity | 5.8 | 0.31 |
| Atrial fibrillation | 2.4 | 0.13 |
| Body mass index | 2.0 | 0.10 |
| Systolic blood pressure | 0.9 | 0.05 |
| Diastolic blood pressure | 0.9 | 0.05 |
| Peripheral vascular disease | 0.7 | 0.04 |
| Triglycerides | 0.5 | 0.03 |
| Fasting glucose | 0.4 | 0.02 |
| Congestive heart failure | 0.2 | 0.01 |
| Myocardial infarction | <0.1 | <0.01 |
| Total | 100% | 5.3% |

Relative importance is expressed as the percentage of the model R² attributable to each variable, quantified by the Lindeman, Merenda, and Gold (LMG) method and rescaled to sum to 100%. Absolute variance explained is the product of relative importance and the total model R². The model, a multivariable linear regression of log2-transformed, standardized circulating CXCL10 on the variables listed, explained 5.3% of the total variance in CXCL10 (R² = 0.053). CXCL10 = C-X-C motif chemokine ligand 10; LDL = low-density lipoprotein; HDL = high-density lipoprotein.

**Supplementary Table 7.** Bootstrap Inclusion Frequency of All 60 Immune Markers and Their Demographics-Adjusted Associations with Incident Stroke

| **Rank** | **Marker** | **Bootstrap inclusion frequency, %** | **HR per 1 SD** | **Nominal p** | **FDR q** |
| --- | --- | --- | --- | --- | --- |
| **1** | **CXCL10 (IP-10)†** | **76.1** | **1.19** | **0.017** | **0.90** |
| 2 | BDNF | 63.5 | 0.84 | 0.11 | 0.90 |
| 3 | CCL5 (RANTES) | 61.7 | 0.94 | 0.50 | 0.90 |
| **4** | **CXCL9 (MIG)†** | **54.8** | **1.13** | **0.096** | **0.90** |
| 5 | VEGF-D | 53.8 | 1.11 | 0.099 | 0.90 |
| 6 | sFasL | 53.8 | 0.77 | 0.19 | 0.90 |
| 7 | PDGF-BB | 48.6 | 1.01 | 0.88 | 0.95 |
| 8 | IL-1RA | 45.0 | 1.09 | 0.16 | 0.90 |
| 9 | sICAM-1 | 42.7 | 1.15 | 0.11 | 0.90 |
| 10 | CCL11 (eotaxin) | 39.7 | 1.05 | 0.61 | 0.90 |
| 11 | IL-21 | 39.5 | 1.10 | 0.14 | 0.90 |
| 12 | PAI-1 (Serpin E1) | 36.8 | 1.04 | 0.65 | 0.90 |
| 13 | CCL2 (MCP-1) | 32.9 | 1.04 | 0.66 | 0.90 |
| 14 | CSF2 (GM-CSF) | 31.3 | 0.92 | 0.52 | 0.90 |
| 15 | IL-7 | 30.5 | 1.03 | 0.70 | 0.90 |
| 16 | Leptin | 29.1 | 1.05 | 0.62 | 0.90 |
| 17 | IL-12p40 | 27.9 | 1.11 | 0.15 | 0.90 |
| 18 | CXCL1 (GRO-α) | 27.6 | 1.04 | 0.60 | 0.90 |
| 19 | Resistin | 26.4 | 1.05 | 0.58 | 0.90 |
| 20 | IL-12p70 | 24.7 | 0.80 | 0.25 | 0.90 |
| 21 | VCAM-1 | 22.3 | 1.02 | 0.86 | 0.95 |
| 22 | IL-10 | 21.8 | 0.93 | 0.59 | 0.90 |
| 23 | SCF | 20.9 | 0.91 | 0.52 | 0.90 |
| 24 | IL-31 | 20.3 | 0.82 | 0.27 | 0.90 |
| 25 | CCL7 (MCP-3) | 20.2 | 0.85 | 0.20 | 0.90 |
| 26 | β-NGF | 19.4 | 0.77 | 0.13 | 0.90 |
| 27 | IFN-β | 18.7 | 1.01 | 0.87 | 0.95 |
| 28 | IL-4 | 17.2 | 0.97 | 0.81 | 0.95 |
| 29 | VEGF-A | 16.2 | 1.06 | 0.44 | 0.90 |
| 30 | EGF | 15.0 | 0.97 | 0.82 | 0.95 |
| 31 | IL-23 | 15.0 | 0.91 | 0.57 | 0.90 |
| 32 | IL-18 | 14.4 | 1.04 | 0.70 | 0.90 |
| 33 | IL-1α | 13.3 | 1.09 | 0.18 | 0.90 |
| 34 | TNF-α | 11.1 | 0.95 | 0.70 | 0.90 |
| 35 | CSF1 (M-CSF) | 10.5 | 0.91 | 0.63 | 0.90 |
| 36 | IL-5 | 9.6 | 0.88 | 0.42 | 0.90 |
| 37 | CXCL8 (IL-8) | 9.5 | 0.84 | 0.46 | 0.90 |
| 38 | IFN-α2 | 9.0 | 0.94 | 0.66 | 0.90 |
| 39 | CCL3 (MIP-1α) | 8.8 | 0.99 | 0.90 | 0.95 |
| 40 | TRAIL | 8.6 | 1.09 | 0.24 | 0.90 |
| 41 | IL-17F | 8.4 | 0.97 | 0.79 | 0.95 |
| 42 | TNF-β | 7.5 | 0.97 | 0.79 | 0.95 |
| 43 | HGF | 7.3 | 1.05 | 0.48 | 0.90 |
| 44 | IL-1β | 7.1 | 0.91 | 0.61 | 0.90 |
| 45 | TGF-β | 6.7 | 0.83 | 0.42 | 0.90 |
| 46 | CXCL12α (SDF-1α) | 6.6 | 1.07 | 0.37 | 0.90 |
| 47 | TGF-α | 6.6 | 0.95 | 0.71 | 0.90 |
| 48 | IL-22 | 6.4 | 0.99 | 0.96 | 0.97 |
| 49 | IL-13 | 6.2 | 0.92 | 0.63 | 0.90 |
| 50 | LIF | 5.8 | 1.00 | 0.98 | 0.98 |
| 51 | IL-2 | 5.4 | 0.89 | 0.47 | 0.90 |
| 52 | CCL4 (MIP-1β) | 4.8 | 1.06 | 0.42 | 0.90 |
| 53 | IL-27 | 4.2 | 0.91 | 0.68 | 0.90 |
| 54 | IL-17A | 3.1 | 0.99 | 0.94 | 0.97 |
| 55 | FGF-basic | 3.0 | 0.84 | 0.48 | 0.90 |
| 56 | IL-6 | 2.9 | 0.93 | 0.60 | 0.90 |
| 57 | IL-9 | 2.4 | 0.82 | 0.47 | 0.90 |
| 58 | IFN-γ | 2.2 | 0.97 | 0.83 | 0.95 |
| 59 | CSF3 (G-CSF) | 1.5 | 0.86 | 0.37 | 0.90 |
| 60 | IL-15 | 1.4 | 0.95 | 0.75 | 0.94 |

Markers are ordered by bootstrap inclusion frequency, the percentage of 2000 bootstrap resamples in which the marker was selected when the entire LASSO procedure, including cross-validated choice of the penalty, was repeated. Hazard ratios, nominal p values, and FDR q values are from separate Cox models for each marker adjusted for age, sex, and race and ethnicity. The q values were computed with the Benjamini–Hochberg procedure across all 60 tests. Bonferroni-adjusted p values were 1.00 for all markers. †Markers selected by LASSO in the primary analysis (Supplementary Table 1). BDNF = brain-derived neurotrophic factor; CCL = C-C motif chemokine ligand; CSF = colony-stimulating factor; CXCL = C-X-C motif chemokine ligand; FDR = false discovery rate; HR = hazard ratio; LASSO = least absolute shrinkage and selection operator; SD = standard deviation. Other abbreviations are defined in Supplementary Table 1.
