# Supplementary material for: Chemokine CXCL10 and Incident Stroke Risk in the Northern Manhattan Study
Source: Transl Stroke Res. 2026 Oct 2;17(5):107. doi: 10.1007/s12975-026-01498-7 (PMC13633176; doi:10.1007/s12975-026-01498-7)
Supplement: Supplementary file 2 [file 12975_2026_1498_MOESM2_ESM.docx]

**Supplementary Figure 1.** Correlations of Plasma CXCL9 and CXCL10 with Each Other, with Systemic Inflammatory Markers, and with White Matter Hyperintensity Volume

Panel A. CXCL9, CXCL10, and Systemic Inflammatory Markers

Panel B. White Matter Hyperintensity Volume

In Panel A, the left plot shows the correlation between CXCL9 and CXCL10 (N = 1175). The remaining plots show the correlation of each systemic inflammatory marker with CXCL9 (solid blue line) and CXCL10 (dashed orange line), with linear regression lines and 95% confidence bands. Panel B shows the corresponding correlations with white matter hyperintensity volume, normalized to total cranial volume. Coefficients are Spearman rank correlations (ρ) with two-sided p values. Values were log-transformed and standardized for display. Because Spearman correlations depend only on rank order, these transformations do not alter the coefficients. N = 1175 for all markers except hsCRP (N = 718). CXCL9 = C-X-C motif chemokine ligand 9; CXCL10 = C-X-C motif chemokine ligand 10; hsCRP = high-sensitivity C-reactive protein; IL-1β = interleukin-1β; IL-6 = interleukin-6; TNF-α = tumor necrosis factor-α.

**Supplementary Figure 2.** Association of Baseline Plasma CXCL9 with Subsequent Stroke as Depicted by Restricted Cubic Spline Analysis

Hazard ratios were estimated with a restricted cubic spline with four knots in a Cox model adjusted for the global vascular risk score. CXCL9 is expressed as z-standardized median fluorescence intensity, and the reference value is the median. The shaded area represents the 95% confidence interval. CXCL9 = C-X-C motif chemokine ligand 9.

**Supplementary Figure 3.** Association of Baseline Plasma CXCL10 with Subsequent Stroke as Depicted by Restricted Cubic Spline Analysis

Hazard ratios were estimated with a restricted cubic spline with four knots in a Cox model adjusted for the global vascular risk score. CXCL10 is expressed as z-standardized median fluorescence intensity, and the reference value is the median. The shaded area represents the 95% confidence interval. CXCL10 = C-X-C motif chemokine ligand 10.
