# Supplementary material for: Chemokine CXCL10 and Incident Stroke Risk in the Northern Manhattan Study
Source: Transl Stroke Res. 2026 Oct 2;17(5):107. doi: 10.1007/s12975-026-01498-7 (PMC13633176; doi:10.1007/s12975-026-01498-7)
Supplement: Supplementary file 3 [file 12975_2026_1498_MOESM3_ESM.docx]

**Supplementary Methods**

*Assessment of Nonlinearity*

To evaluate departures from linearity in the associations of circulating biomarkers with incident stroke, three complementary approaches were applied within Cox proportional hazards models adjusted for the global vascular risk score (GVRS). Biomarkers were analyzed as binary variables split at the cohort median and as quartiles, which allow a distribution-free assessment of risk gradients. Restricted cubic splines with four knots at standard percentiles characterized the shape of each association without assuming a functional form. Quadratic terms added to models containing the linear term provided a formal test for curvature. Linear terms were considered adequate when these approaches consistently showed no evidence of nonlinearity.

*Determinants of Variance in Circulating CXCL10*

Determinants of variance in plasma CXCL10 levels were examined in a multivariable linear model of log2-transformed, standardized CXCL10 on age, sex, race and ethnicity, body mass index, systolic and diastolic blood pressure, low-density lipoprotein (LDL) cholesterol, high-density lipoprotein (HDL) cholesterol, triglycerides, fasting glucose, peripheral vascular disease, atrial fibrillation, myocardial infarction, congestive heart failure, white matter hyperintensity volume, and estimated glomerular filtration rate. Relative importance was quantified by the Lindeman, Merenda, and Gold decomposition of the model R², implemented in the relaimpo package in R and rescaled to sum to 100% across variables (Supplementary Table 6).

*Accounting for Data-Driven Marker Selection*

Selecting markers and testing them in the same data yields p values that do not reflect the uncertainty of the selection step [S1]. Two complementary analyses addressed this issue. The entire LASSO procedure was repeated in each of 2000 nonparametric bootstrap resamples of the analytic sample. In each resample, the penalty was chosen by fivefold cross-validation with randomly assigned folds, and markers with nonzero coefficients at λ_min_ were recorded. The bootstrap inclusion frequency of each marker was the proportion of resamples in which it was selected [S2, S3]. This approach follows the principle of stability selection, in which variables selected consistently under resampling are regarded as robust to sampling variability [S4]. Each of the 60 markers was also entered in a separate Cox model adjusted for age, sex, and race and ethnicity. Nominal p values were corrected across all 60 tests with the Benjamini–Hochberg false discovery rate (FDR) procedure [S5] and with the Bonferroni method. Analyses used the glmnet, survival, and parallel packages in R (Supplementary Table 7).

**Supplementary Results**

*Assessment of Nonlinearity and Alternative Functional Forms*

In secondary analyses, point estimates for above-median versus below-median CXCL9 and CXCL10 indicated greater stroke risk, but none of these associations reached statistical significance in demographics-adjusted or GVRS-adjusted models (Supplementary Table 3).

Quartile analyses showed graded associations for both chemokines in GVRS-adjusted models that did not reach statistical significance (per quartile, CXCL9 HR 1.17; 95% CI 0.99–1.38; CXCL10 HR 1.15; 95% CI 0.98–1.34). For CXCL9, the highest versus lowest quartile contrast showed borderline evidence of greater risk (Q4 vs. Q1 HR 1.61; 95% CI 0.96–2.72). For CXCL10, point estimates suggested greater risk in Q2 to Q4 than in Q1, although none of these contrasts was statistically significant (Supplementary Table 4).

Restricted cubic spline analyses showed no evidence of nonlinearity for either biomarker (CXCL9, χ² = 3.44, df = 2, p = 0.18; CXCL10, χ² = 1.44, df = 2, p = 0.49). Consistent with this, quadratic terms were not statistically significant for either marker, providing no evidence of U-shaped or J-shaped associations. Model-based predicted hazard ratio curves supported an approximately linear relationship across the observed range (Supplementary Figures 2 and 3).

**Supplementary References**

S1. Berk R, Brown L, Buja A, Zhang K, Zhao L: Valid post-selection inference. The Annals of Statistics 2013, 41(2):802-837.

S2. Sauerbrei W, Schumacher M: A bootstrap resampling procedure for model building: application to the Cox regression model. Statistics in Medicine 1992, 11(16):2093-2109.

S3. Heinze G, Wallisch C, Dunkler D: Variable selection – A review and recommendations for the practicing statistician. Biometrical Journal 2018, 60(3):431-449.

S4. Meinshausen N, Bühlmann P: Stability selection. Journal of the Royal Statistical Society Series B: Statistical Methodology 2010, 72(4):417-473.

S5. Benjamini Y, Hochberg Y: Controlling the false discovery rate: a practical and powerful approach to multiple testing. Journal of the Royal Statistical Society Series B: Statistical Methodology 1995, 57(1):289-300.
